# Interstitial lung disease in STING-associated vasculopathy with onset in infancy (SAVI): preliminary genotype-phenotype correlation

**DOI:** 10.1186/1546-0096-13-S1-O32

**Published:** 2015-09-28

**Authors:** L Malle, B Marrero, Y Liu, G Montealegre, D Chapelle, H Kim, M O'Brien, S Hill, JR Fontana, S Ramsey, G Duckers, S Ozen, A Issekutz, H Wittkowski, D Foell, K Tenbrock, O Jones, S Holland, B Gonzalez, P Brogan, E Omoyinmi, S Melo Gomes, A Paller, Z Deng, R Goldback-Mansky, A Almeida de Jesus

**Affiliations:** 1 National Institutes of Health, Translational Autoinflammatory Diseases Section, Bethesda, USA; 2 National Institutes of Health, NIAMS, Bethesda, USA; 3 National Institutes of Health, Radiology Department, Bethesda, USA; 4 National Institutes of Health, NHLBI, Bethesda, USA; 5 Dalhousie University, Halifax, Canada; 6 HELIOS Klinikum Krefeld, Krefeld, Germany; 7 Hacettepe University Hospitals, Ankara, Turkey; 8 University Hospital of Muenster, Muenster, Germany; 9 University of Germany, Aachen, Germany; 10 US Army, Bethesda, United States; 11 National Institutes of Health, NIAID, Bethesda, USA; 12 Hospital Luiz Calvo Mackenna, Santiago, Chile; 13 ICH, and Great Ormond Street Hospital NHS Foundation Trust, London, UK; 14 Northwestern University Feinberg School of Medicine, Chicago, USA

## Background

Some monogenic interferonopathies are caused by innate immune dysregulation and form a subclass of autoinflammatory disorders characterized by systemic inflammation due to chronic Type I interferon stimulation. STING-Associated Vasculopathy with Onset in Infancy (SAVI) is an IFN-mediated disease caused by gain-of-function mutations in *TMEM173*, the gene encoding the stimulator of interferon genes (STING).

## Objectives

This study was undertaken to understand the variable disease severity of the interstitial lung disease (ILD) in SAVI patients. We hypothesized that the severity of the interstitial lung disease may be modulated by a common SNP (R232H, rs1131769) that is functionally associated with decreased *IFNB1* transcription.

## Methods

We studied nine SAVI patients with N154S, V155M, or V147L mutations. Lung involvement was assessed by chest computed tomography (CT) and pulmonary function tests (PFTs) for all patients, a lung biopsy was available for five patients. Peripheral blood genomic DNA samples were obtained and *TMEM173* (NM_198282.3) was sequenced by Sanger technique. STING function was evaluated in the different *TMEM173* haplotypes by *IFNB1* Luciferase Reporter assays performed with cells transfected with wildtype or mutant *TMEM173* on the R232 and the H232 backgrounds.

## Results

We described the clinical features of ILD in nine SAVI patients. Six patients had evidence of severe ILD characterized by moderate to severe abnormalities on chest CT, PFTs and/or lung biopsy. Two patients presented with mild ILD and one did not have any evidence of ILD. Four out of the six patients with severe ILD succumbed to pulmonary complications. Five patients with severe ILD were homozygous for R232 (R232/R232) and one was heterozygous for the SNP. Conversely, the two patients with mild ILD were heterozygous (R232/H232) and the patient without ILD was homozygous for the H232 allele (H232/H232). Thus, the severity of interstitial lung disease seems to correlate with the STING haplotype. Transfection of HEK293T cells with the H232 *TMEM173* haplotype with or without SAVI causing mutations results in decreased *IFNB1* expression in the presence of both low affinity and high affinity STING stimulator cGAMP in comparison with cells transfected with the R232 haplotype. These findings suggest that the H232 haplotype background may be protective from the development of ILD.

## Conclusion

The variable presentation and severity of ILD in SAVI patients seems to correlate with the *TMEM173* haplotype at position 232 and possibly with the local induction of an IFN response. Our data suggest that common variants can modify disease expression specific to one organ and provide a model to assess the variable disease phenotype in other interferonopathies.

